# Using genetic and phenetic markers to assess population isolation within the southernmost tsetse fly belt in Africa

**DOI:** 10.4102/ojvr.v86i1.1768

**Published:** 2019-10-16

**Authors:** Chantel J. De Beer, Gert J. Venter, Marc J.B. Vreysen, Fernando C. Mulandane, Luis Neves, Sihle Mdluli, Otto Koekemoer

**Affiliations:** 1 Department of Epidemiology, Parasites and Vectors, Agricultural Research Council, Onderstepoort Veterinary Research (ARC-OVR), Pretoria, South Africa; 2 Department of Veterinary and Tropical Diseases, Faculty of Veterinary Science, University of Pretoria, Pretoria, South Africa; 3 Insect Pest Control Laboratory, Joint FAO/IAEA Division of Nuclear Techniques in Food and Agriculture, Seibersdorf, Austria; 4 Biotechnology Centre, Eduardo Mondlane University, Maputo, Mozambique; 5 Department of Veterinary Services, Epidemiology Unit, Mbabane, Eswatini

**Keywords:** *Glossina brevipalpis*, *Glossina austeni*, geometric morphometrics, mitochondrial DNA, veterinary entomology

## Abstract

The effective control of tsetse flies (Diptera; Glossinidae), the biological vectors of trypanosome parasites that cause human African trypanosomosis and African animal trypanosomosis throughout sub-Saharan Africa, is crucial for the development of productive livestock systems. The degree of genetic isolation of the targeted populations, which indicate reinvasion potential from uncontrolled areas, will be critical to establish a control strategy. Molecular and morphometrics markers were used to assess the degree of genetic isolation between seemingly fragmented populations of *Glossina brevipalpis* Newstead and *Glossina austeni* Newstead present in South Africa. These populations were also compared with flies from adjacent areas in Mozambique and Eswatini. For the molecular markers, deoxyribonucleic acid was extracted, a r16S2 Polymerase chain reaction (PCR) was performed and the PCR product sequenced. Nine landmarks were used for the morphometrics study as defined by vein intersections in the right wings of female flies. Generalised Procrustes analyses and regression on centroid size were used to determine the Cartesian coordinates for comparison between populations. Both methods indicated an absence of significant barriers to gene flow between the *G. brevipalpis* and *G. austeni* populations of South Africa and southern Mozambique. Sustainable control can only be achieved if implemented following an area-wide management approach against the entire *G. brevipalpis* and *G. austeni* populations of South Africa and southern Mozambique. Limited gene flow detected between the *G. austeni* population from Eswatini and that of South Africa or Mozambique may imply that these two populations are in the proses of becoming isolated.

## Introduction

African animal trypanosomosis is endemic in South Africa with an estimated 350 000 cattle at risk in the rural north east of the KwaZulu-Natal province (Kappmeier, Nevill & Bagnall 1998; Kappmeier Green, Potgieter & Vreysen 2007). The sustainable control of tsetse flies (Diptera; Glossinidae), the vectors of the trypanosome parasites (Kinetoplastida; Trypanosomatidae), therefore remains a priority.

Presently, two species of tsetse flies, *Glossina brevipalpis* Newstead and *Glossina austeni* Newstead, are found in an area of about 16 000 km^2^ in the north eastern part of the KwaZulu-Natal province in South Africa. The infested area stretches from about 10 km south of the Mfolozi River in the south, for approximately 190 km, to the border of Mozambique in the north, and from the Indian Ocean coast in the east for 80 km to the Hluhluwe-iMfolozi Park in the west (De Beer et al. 2016a; Kappmeier Green et al. 2007).

These South African populations of *G. austeni* and *G. brevipalpis*, extending into the Matutuine district (Maputo Province) of Mozambique, represent the southern most distribution of tsetse flies in Africa (Sigauque et al. 2000). The Matutuine district borders in the south with the north eastern part of the KwaZulu-Natal province in South Africa. The northern limit is the Boane and Namaacha districts of Mozambique. In the east, it borders with the Indian Ocean and in the west with Eswatini. While, to date, no *G. brevipalpis* has been sampled in Eswatini, low numbers of *G. austeni* were trapped in 2008. These flies were only found in the Mlawula Nature Reserve, situated in the north east of the country (Saini & Simarro 2008). This reserve extends into the Mbuluzi Game Reserve in the north west, the Simunye Nature Reserve in the south and Hlane Royal National Park further to the south west. In the east, the reserve borders with the Lebombo Mountains (elevation 776 m), an 800-km-long narrow range of mountains that stretch from Hluhluwe in the KwaZulu-Natal province in the south to Punda Maria in the Limpopo province in South Africa in the north parallel with the Mozambican border.

Determining the exact geographical limits of tsetse fly populations and the level of genetic interactions between these populations on mainland Africa remains challenging. In contrast to previous surveys in South Africa that indicated *G. brevipalpis* to be confined to two distinct bands in the north and south of the infested area in the KwaZulu-Natal province (Kappmeier Green et al. 2007), thorough surveys (De Beer et al. 2016a) showed that both *G. brevipalpis* and *G. austeni* appear to be more continuously distributed throughout the area. In view of its relatively low abundance in the area between the two bands, it would be important to elucidate the genetic distance between the *G. brevipalpis* populations in the north and the south to develop an appropriate control strategy.

Similarly, the low abundance and patchy distribution of *G. austeni* in this area may result in the development of localised genetically isolated populations. Although *G. austeni*, and perhaps also *G. brevipalpis*, is continuously distributed throughout north eastern KwaZulu-Natal province, these populations are, on a micro-ecological scale, often confined to pockets of dense vegetation (Esterhuizen 2007). This, in addition to the relatively sedentary behaviour of the species, may favour the development of potential genetically isolated populations. It is known that habitat fragmentation can lead to slight but significant variations in localised environmental conditions to which tsetse populations will adapt physiologically and demographically thereby affecting tsetse–trypanosome interactions and hence trypanosomosis risk (Mweempwa et al. 2015).

Eradication of a tsetse population will in most cases only be sustainable if the control programme follows an area-wide integrated pest management (AW-IPM) approach, that is, a strategy that targets the entire pest population (Hendrichs et al. 2005; Klassen 2005). Such an approach is fairly straightforward if the targeted population represents a geographically well-defined population with no gene flow from neighbouring populations, or if a rolling-carpet approach is implemented (Hendrichs et al. 2005). The rolling-carpet approach is dynamic as the basic operational phases (pre-intervention, population reduction, release of sterile insects and maintenance of pest status) are carried out simultaneously in a phased manner (Hendrichs et al. 2005).

Kappmeier Green et al. (2007) proposed an AW-IPM strategy that included a sterile insect technique (SIT) component based on distribution data of the fly populations prior to 1999 (Kappmeier Green 2002). The recent sampling of *G. brevipalpis* in areas where they were considered absent before requires a revisit of the proposed strategy. In the present study, available molecular and phenetic (geometric morphometrics) markers were used to determine the level of genetic isolation between the southern and northern tsetse fly pockets in South Africa. In addition, the South African populations were compared with flies collected in the adjacent areas in southern Mozambique and Eswatini. The level of genetic isolation between the various populations within South Africa and between these three countries will provide an approximation of the invasion potential of flies from neighbouring areas (Solano et al. 2010) into the controlled areas. This knowledge will be a prerequisite for the development of the most appropriate control strategy, that is, whether these populations can be tackled separately or in sequence or whether they constitute one population.

## Materials and methods

### Tsetse fly sampling

**FIGURE 1:**
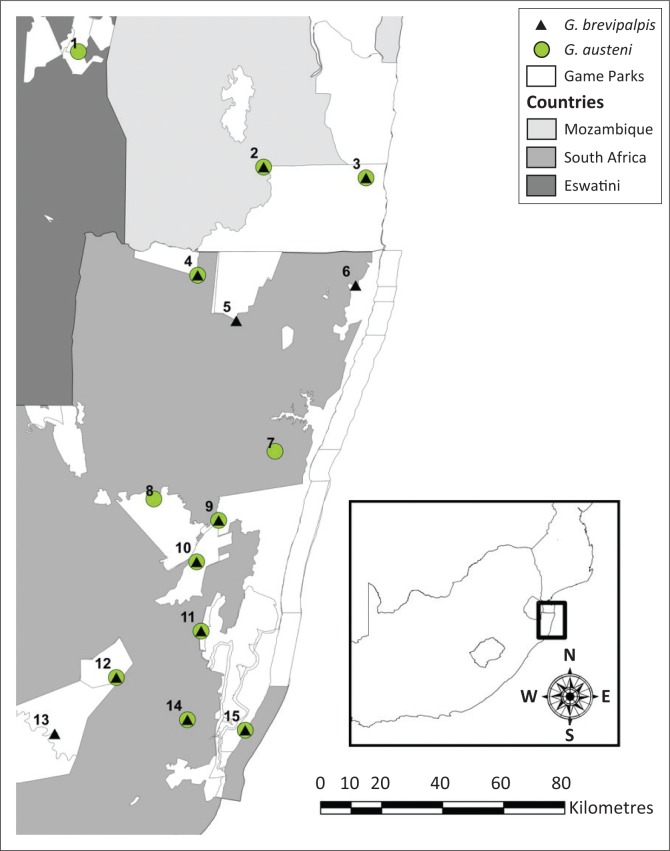
Sites used for the collection of tsetse flies for comparative molecular and geometric morphometrics in Eswatini: (1) Mlawula Nature Reserve, Mozambique, (2) west and (3) east of the Maputo River and South Africa (4) Ndumu, (5) Tembe, (6) Kosi Bay, (7) Mbazana, (8) Mkuzi, (9) Lower Mkuzi, (10) Phinda, (11) False Bay Park, (12) Hluhluwe-iMfolozi Park north, (13) Hluhluwe-iMfolozi Park south, (14) Boomerang and (15) St Lucia. At sites 8, 13 and 21 flies were used only for molecular analyses.

Tsetse flies were collected with odour-baited H-traps (Kappmeier 2000; Kappmeier & Nevill 1999; Kappmeier Green et al. 2007) in 15 sites for molecular marker analyses and in 12 sites for morphometric marker analyses (Figure 1). In South Africa, flies were sampled in 12 and 10 sites for the molecular and morphometric marker analyses, respectively (Figure 1). In southern Mozambique, flies were collected in two sites, east and west of the Maputo River, for molecular marker analyses (Figure 1). Only flies collected east of the Maputo River were subjected to morphometric analyses (Figure 1). In Eswatini, flies were collected only in the Mlawula Nature Reserve as they are confined to this area (Figure 1). These sites comprised the entire geographical distribution area of tsetse flies in the region. In the traps, flies were guided to plastic collection bottles that contained a 20% ethanol solution to which Savlon® (Johnson & Johnson, Pharmedica Laboratories [Pty] Ltd., East London, South Africa) (0.4 mL/L) and formalin (0.4 mL/L) were added. The sampled flies were removed from the collection bottles and preserved in 95% ethanol until analysed.

*Glossina austeni* and *G. brevipalpis* from laboratory colonies maintained at the Agricultural Research Council-Onderstepoort Veterinary Research (ARC-OVR), Pretoria, South Africa, were included for the genetic analyses as out-groups. The colonies of *G. austeni* and *G. brevipalpis* at the ARC-OVR originate from laboratory colonies at the Vector and Vector-Borne Diseases Research Institute, Tanga, Tanzania, and the FAO/IAEA Insect Pest Control Laboratory in Seibersdorf, Austria, respectively (De Beer, Venter & Vreysen 2016b). The colonies in Tanga and Seibersdorf were originally established from material collected in 1982 on the Unguja Island of Zanzibar and Kenya in East Africa (De Beer et al. 2016b). As such these out-groups represent genetically isolated populations which were bred in isolation for nearly 36 years.

### Deoxyribonucleic acid extraction

Deoxyribonucleic acid (DNA) was extracted from ethanol preserved *G. brevipalpis* and *G. austeni* specimens. After removal from the ethanol, the flies were briefly air-dried and rehydrated in phosphate buffered saline (PBS). The wings and legs of the flies were removed, and the body crushed in fresh PBS using a micro-pestle in a 1.5 mL polypropylene tube. After centrifugation, 200 *µ*L of the supernatant suspension was used for DNA extraction with a DNeasy Blood & tissue kit (Qiagen, Johannesburg, South Africa). The purified DNA was suspended in 100 *µ*L of the supplied elution buffer and either processed immediately or stored at −20 °C.

### Mitochondrial locus amplification and sequencing

The N1-J-12585 [5′GGT CCC TTA CGA ATT TGA ATA TAT CCT 3′] and LR-N-12866 [5′ACA TGA TCT GAG TTC AAA CCG G3′] primer pairs (Simon et al. 1994) were used to amplify the mitochondrial 16S-2 ribosomal ribonucleic acid (rRNA) locus from 2 to 5 *µ*L of the extracted DNA as described previously (Krafsur, Marquez & Ouma 2008). The amplification products were verified by gel electrophoresis and sequenced using the same primers as those used for the amplification. The sequencing reactions contained around 10 ng of template DNA and 3.2 pmol of the primer made up to a final volume of 12 *µ*L with ultra-pure H_2_O. Sequencing was performed on a 3100 Genetic Analyser (Applied Biosystems, South Africa (Pty) Ltd, Johannesburg) using the Big Dye Terminator V 3.1 Cycle Sequencing Kit (Applied Biosystems, South Africa (Pty) Ltd).

### Morphometric analysis

**FIGURE 2:**
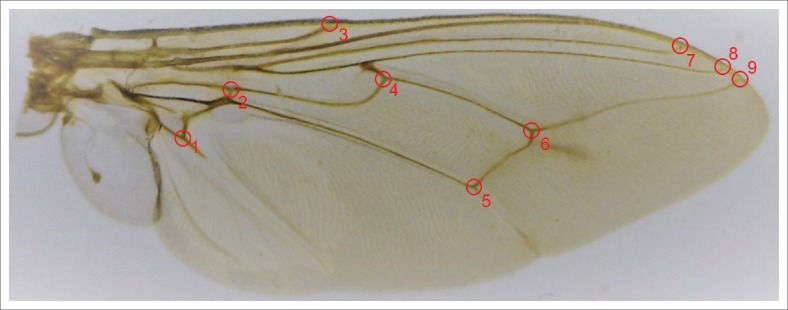
*Glossina austeni* slide-mounted wing indicating the nine landmarks as defined by vein intersections.

For morphometric analysis, the right wings of 40 females of each species per site were used. Lower numbers of flies were available from Mozambique (*n* = 36), Kosi Bay (*n* = 31) and False Bay Park (*n* = 37) for *G. brevipalpis* and Mozambique (*n* = 14) and the Hluhluwe-iMfolozi Park (*n* = 13) for *G. austeni*. The right wings of 345 *G. brevipalpis* and 346 *G. austeni* females were removed, dry mounted between two microscope slides (Patterson & Schofield 2005) and photographed using a Dino X Lite Digital Microscope (IDCP B.V, Naarden, The Netherlands). Nine landmarks (Cartesian coordinates) were defined by vein intersections (Figure 2) using the Collection of Coordinates (COO) programme of the Collection of Landmark for Identification and Characterisation (CLIC) software package (Dujardin, Kaba & Henry 2010).

### Data analysis

Deoxyribonucleic acid sequences were aligned with Clustal Omega (Sievers et al. 2011), keeping all default parameters. The alignment files were exported in FASTA format and used as input for MEGA7 (Kumar, Stecher & Tamura 2016) to construct the phylogenetic trees and IBDWS (Jensen, Bohonak & Kelley 2005) to estimate the fixation index (Fst values) from haploid genetic distance data, according to the methods used by Weir (1990). Gaps at the end of sequences were treated as missing data. Phylogenetic trees were constructed from the aligned sequences using the maximum likelihood algorithm with a substitution model and 500 bootstrap replications (Tamura & Nei 1993). The model of nucleotide substitution that best fits the data set was determined using the maximum likelihood model selection function in MEGA7 software (Kumar et al. 2016). Separate trees, using 16S rRNA-gene sequence data, were generated for each species.

Morphometric analyses of the Cartesian coordinates were carried out using the MorphoJ integrated software package (Klingenberg 2011). The Cartesian coordinates were subjected to a generalised Procrustes analysis (Rohlf 1996) and variations in wing shape (partial warps) were determined by Procrustes superposition through generalised least squares (Rohlf 1999). Through Procrustes superimposition analyses, the wings were scaled to the same size, transposed to the same centre of gravity and orientated to provide the minimum sum of squared distances between corresponding landmarks. Principal component analyses of the shape variables using MorphoJ (Klingenberg 2011) provided 14 partial warps. Differences in wing shape were determined by Canonical shape dissimilarity (Klingenberg 2011).

In addition, the centroid size, as an indicator of size variation, was determined by the square root of the sum of the squared distances of all landmarks from the centroid (Bookstein 1991). Centroid size was analysed using the statistical software GraphPad Instat (Version 3.00, 2003). A one-way analysis of variance (ANOVA) was used to assess significant differences between the means of the centroid size (*p* value < 0.05 was considered significant) of wings of flies collected from the various sites. The data were normally distributed, standard (parametric) methods were used and the Tukey’s test was applied. Multivariate regression of partial warps on size was used to estimate the residual allometry and the statistical significance was estimated by the 10 000 runs permutation tests (Klingenberg 2011).

### Ethical considerations

Materials used in the study posed no health risk to researchers and no vertebrate animals were involved. The study was conducted as part of a project funded by the Department of Science and Technology on National Assets (P10000035) at the ARC-OVR (Agricultural Research Council, Onderstepoort Veterinary Research) in collaboration with the Food and Agriculture Organization (FAO) or International Atomic Energy Agency (IAEA) Division of Nuclear Techniques in Food and Agriculture and the Department of Technical Cooperation of the IAEA under project RAF 5069. Permission to carry out research in terms of Section 20 of the *Animal Diseases Act* South Africa (Act no. 35 of 1984) has been granted for tsetse fly collection (Ref 12/11/1/1/9).

## Results

### Mitochondrial deoxyribonucleic acid analysis

**FIGURE 3:**
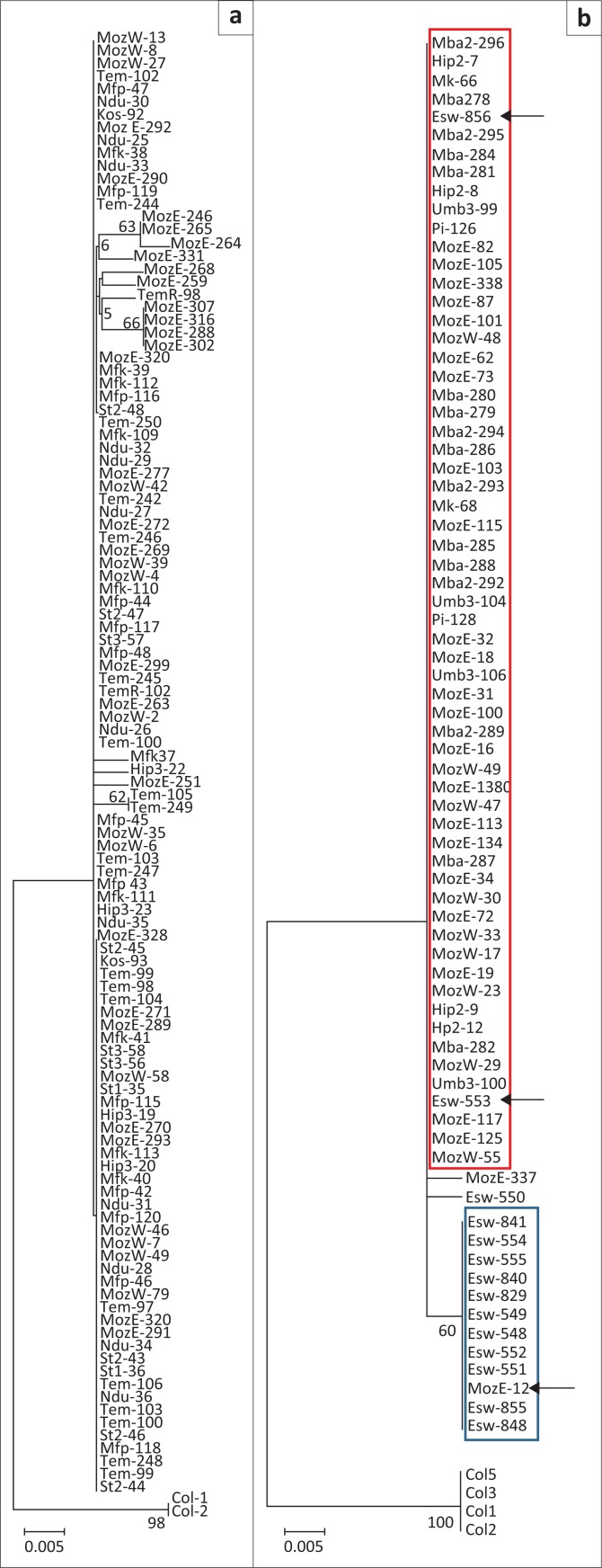
Phylogenetic trees constructed from partial sequence analysis of the 16S subunit 2 ribosomal ribonucleic acid gene of *Glossina brevipalpis* (a) and *Glossina austeni* (b) from Eswatini (Esw), Mozambique (Moz E; Moz W) and South Africa [Ndumu (NDU), Tembe (TEM), Kosi Bay (KOS), Mbazana (MBA), Mkuzi (MK), Lower Mkuzi (UMB), Phinda (PI), False Bay Park (MFP), Hluhluwe-iMfolozi Park north (HIP), Hluhluwe-iMfolozi Park south (HP), Boomerang (MFK), St Lucia (ST)]. Colony (Col) flies of both species were used as out-groups. Red and blue groupings indicate the South Africa-southern Mozambique and Eswatini haplotypes, respectively. Arrows indicate specimens carrying contrasting haplotypes from their group.

After quality checks, mitochondrial DNA (mtDNA) sequences from 79 *G. austeni* and 116 *G. brevipalpis* specimens were used in multiple alignments, which included sequences from four *G. austeni* and two *G. brevipalpis* from the tsetse colonies as out-groups. The phylogenetic trees (Figure 3), constructed from the aligned sequences, showed minimum variation.

The phylogeny of *G. brevipalpis* from South Africa and southern Mozambique did not show any significant branching or grouping of specimens, with the exception of the colony flies (Figure 3a). For both species, a prominent Mozambique South Africa genotype was evident with only minor substructuring of isolates. The branches or groupings did not correlate with geographic origin and the nodes had very low bootstrap support values (*p*-distances < 0.01). The only exceptions were the colony flies and *G. austeni* from Eswatini which formed a distinct phylogenetic grouping (Figure 3b). The Eswatini *G. austeni,* however, did not form a monophyletic group as two specimens carried the South Africa-southern Mozambique haplotype and one specimen (Esw-550) had a unique haplotype (Figure 3b). Additionally, one specimen from southern Mozambique (Moz E-12) carried the Eswatini haplotype (Figure 3b). For *G. austeni*, 198 sites out of 349 in the sequence alignments were variable and the haplotypic diversity was 0.106 overall, and 0.054 when the out-group consisting of the colonised East African specimens was excluded.

The Fst values indicate a great level of genetic differentiation between the *G. austeni* populations from South Africa and Eswatini (0.22), whereas the level of genetic differentiation was moderate between the populations of southern Mozambique and Eswatini (0.15).

### Morphometric analysis

The shape of the right wings of female *G. brevipalpis* and *G. austeni* was analysed to assess the degree of genetic isolation between the populations within South Africa as well as between the South African and the southern Mozambique populations. The analysis for the *G. austeni* populations was extended to include the population in Eswatini. The allometric effect was removed so that shape could be analysed independently.

**FIGURE 4:**
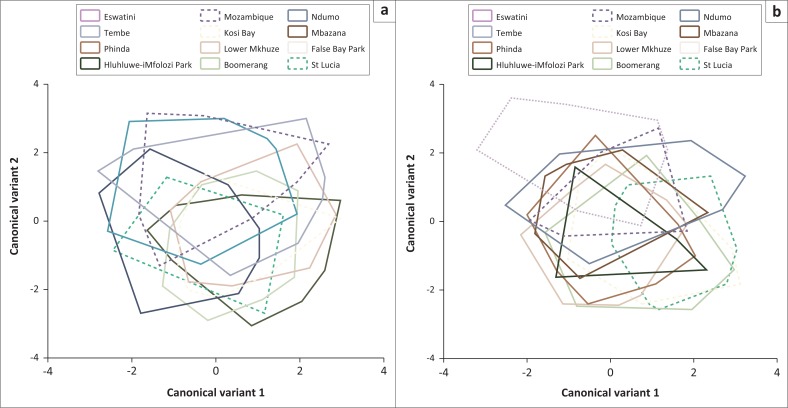
The distribution of *Glossina brevipalpis* (a) and *Glossina austeni* (b) female right wing shape in the morphospace defined by the first two Canonical variants; flies were collected from different sites.

The two species were evaluated separately. The principal component analyses indicated that the first two discriminant factors (shape components) accounted for 71% and 69% of the variance for *G. brevipalpis* and *G. austeni*, respectively. These discriminant factors indicated that there was no clear wing shape separation between the *G. brevipalpis* populations collected from the sites in South Africa and southern Mozambique (Figure 4a). The multivariate regression of the first relative warp against centroid size (100 000 permutation rounds) was also not significant (*p* = 0.30), indicating that there was no residual allometry.

In contrast, the observed residual allometry for *G. austeni*, as the multivariate regression was significant (*p* = 0.01), might indicate an environmental effect. There was no separation within the first discriminant factors between the *G. austeni* collected from the different sites in South Africa as well as those from southern Mozambique (Figure 4b). In addition, no shape separation was observed between *G. austeni* collected from Eswatini, southern Mozambique or South Africa (Figure 4b). The only exception includes flies collected at St Lucia and Eswatini. This is explained by the 228 km distance between these two sites, located at the boundaries of the study area (Figure 1).

**FIGURE 5:**
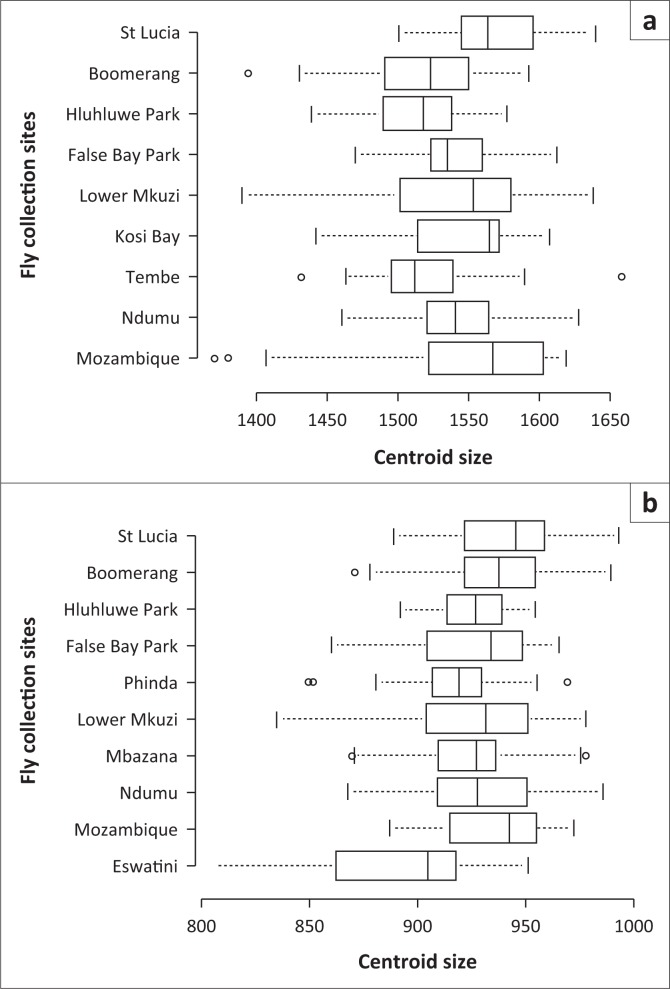
Centroid size variations of the right wings of female *Glossina brevipalpis* (a) and *G. austeni* (b) according to localities. Each box shows the group median separating the 25th and 75th quartiles, the capped bars indicate maximum and minimum values and circles indicate the outliers. Boxes followed by a different letter indicate that the centroid size was significantly different at the 5% level.

The wing size variation as indicated by the isometric estimator known as the average wing centroid size was determined for both species. For *G. brevipalpis*, this ranged from 1512 ± 35 for flies collected from the Hluhluwe-iMfolozi Park to 1568 ± 35 for flies from St Lucia (Figures 1 and 5a). The most significant differences in wing centroid size were found between the populations of the Hluhluwe-iMfolozi Park and southern Mozambique (*p* < 0.01), Ndumu (*p* < 0.05), Kosi Bay (*p* < 0.05) and St Lucia (*p* < 0.01) (Figure 5a). Significant differences in wing centroid size were also observed between the *G. brevipalpis* populations sampled in southern Mozambique and Boomerang (*p* < 0.05), Tembe versus St Lucia (*p* < 0.01) and Boomerang versus St Lucia (*p* < 0.01) (Figure 5a).

Within South Africa, wing centroid size was significantly different (*p* < 0.01) between *G. austeni* from Phinda (916 ± 26) and St Lucia (941 ± 52) (Figure 5b). Similarly, the average female wing centroid size of the *G. austeni* population from Eswatini (892 ± 37) was significantly different (*p* < 0.01) from that of the flies from southern Mozambique and South Africa (Figure 5b).

This indicates a longitudinal (coast to interior) trend in wing size for both species. Flies collected at the coastal sites were, on average, bigger than flies collected in the interior (Figure 5). A trend in wing size, although to a lesser extent, was also observed in a latitudinal direction with bigger flies, on average, collected in the south (Figure 5).

## Discussion

The southern Mozambique populations of *G. brevipalpis* and *G. austeni* extend into South Africa and Eswatini (*G. austeni*) (Saini & Simarro 2008; Sigauque et al. 2000). The confinement of flies to areas with suitable vegetation (i.e. protected areas and game parks) linked to their relatively sedentary behaviour and habitat fragmentation in the area might have created opportunities for these populations to become genetically isolated.

Molecular and/or morphometric markers have been used in the past to estimate gene flow between subpopulations as an indirect measure of dispersal (Bouyer et al. 2007, 2009; Camara et al. 2006; Gooding & Krafsur 2005; Solano et al. 2000, 2009). Geometric wing morphometry in conjunction with microsatellite analyses showed that the populations of *Glossina palpalis gambiensis* that Vanderplank found on the Loos Islands, 5 km off the coast of Guinea, were genetically isolated from two populations on mainland Guinea (Camara et al. 2006). Similarly, a study using microsatellite and mtDNA markers in conjunction with morphometrics showed that the *G. p. gambiensis* population of the Niayes in Senegal was genetically isolated from the main tsetse belt 120 km away in the eastern part of the country (Solano et al. 2010).

The mtDNA sequences as obtained with the 16srRNA gene marker used in the present study were sensitive enough to discriminate between *G. austeni* populations from South Africa-southern Mozambique and those of Eswatini. There was, however, low variability within and between tsetse fly populations of both species from South Africa and southern Mozambique. The presence of a South Africa-southern Mozambique haplotype specimen collected from Eswatini and vice versa (two specimens) indicate limited gene flow and potential incomplete isolation (Figure 3). This isolation might be driven by the geographical distance between the sites, the physical barrier represented by the Lebombo Mountains and regular bush fires occurring in the Mlawula Nature Reserve in Eswatini.

Although the Fst values were potentially skewed by the unequal numbers in each of the populations, the phylogenetic analysis seems to indicate that there is probably more migration of *G. austeni* between Eswatini and southern Mozambique than between Eswatini and South Africa, which is plausible, given the locations and geography of the sampling sites (Figure 1).

Morphometric markers after Procrustes superposition indicated a lack of any significant differentiation in wing shape between populations within South Africa, southern Mozambique and Eswatini. In congruence with the molecular analyses, wing shape variations indicated that the *G. austeni* from Eswatini was to some extent isolated from the South Africa-southern Mozambique populations.

The significant differences obtained in wing centroid size for both species collected at various sites within South Africa as well as in neighbouring countries are the result of variation in environmental condition between sites. As indicated by De Beer (2016), the temperature and humidity fluctuations were more pronounced in the interior, for example, Ndumu than at the coast, for example, St Lucia. The mean temperature in the interior ranged from 27 °C in the hot months to 15.5 °C in the colder months, and the mean relative humidity from 80% in the rainy to 50% in the dry season (De Beer 2016). The mean temperature at the coast ranged from 26 to 17 °C and the mean relative humidity from 99% to 68% (De Beer 2016). This indicates that populations at the coast are subjected to less pronounced fluctuations in environmental conditions than individuals collected in the interior.

Although comparable results were obtained with morphometric analyses and those using mtDNA, both methods have limitations. The use of microsatellite markers, which have proved to be very useful and accurate to study isolation between populations of the same tsetse species (Kaba et al. 2012), was not possible as these markers were not developed at the time of the study. We may therefore have missed out the information on real isolation between populations that may have occurred recently, because of a lack of variability and, hence, sensitivity of the 16srRNA gene marker. In the evaluation of these results, it must be taken into consideration that mtDNA is a very slow evolving fragment of the DNA (Galtier et al. 2009) and that the lack of differentiation, as obtained in the present study, could be because of low sensitivity of the marker chosen. In addition, the marker is maternally inherited and may or may not reflect patterns of nuclear differentiation (Galtier et al. 2009). The current data prompt a more conclusive analysis, using microsatellites, which is currently underway, to confirm or refute the present results.

It still needs to be confirmed whether the tsetse populations that encompass South Africa, southern Mozambique and Eswatini are isolated from the main tsetse fly belt north of Maputo. It is assumed that this belt starts approximately 500 km further north in central Mozambique south of the Save River (Dias 1961; Mulandane 2013). The presence of *Trypanosoma congolense* positive cattle, diagnosed during routine surveillance programmes in the area between Maputo and the Save River, is a strong indication of the presence of tsetse flies as cyclical vectors. This area needs to be surveyed in detail to confirm the presence or absence of tsetse files. Should flies be sampled, these need to be included in the planned genetic study using microsatellites. The degree of genetic isolation of the southern African population from those further north in Africa also needs to be determined.

Within the known limitations of the used markers, the data of this study seem to provide some evidence of the absence of significant barriers to gene flow between the populations in South Africa and southern Mozambique and justify the need for further investigation. This seems to imply that the *G. brevipalpis* and *G. austeni* populations of South Africa and southern Mozambique can be considered homogenous and that localised control (e.g. only in South Africa) may not be sustainable because of reinvasion from uncontrolled neighbouring areas. Should the data be confirmed by the ongoing microsatellite work, this would entail the need for an AW-IPM approach against the entire tsetse fly belt of South Africa and southern Mozambique. The entire area should be controlled simultaneously or in a sequential way using temporary barriers of impregnated traps and/or targets between tsetse free areas following the ‘rolling-carpet’ principle (Vreysen et al. 2007).
